# *Croton buiquensis* (Euphorbiaceae), a new species from northeastern Brazil, its phylogenetic placement, and niche modeling

**DOI:** 10.7717/peerj.20718

**Published:** 2026-02-06

**Authors:** Joesili C.P. Oliveira, Karen Y. Suarez-Contento, Ricarda Riina, Sarah M. Athiê-Souza

**Affiliations:** 1 Departamento de Biologia, Programa de Pós-graduação em Biodiversidade, Universidade Federal Rural de Pernambuco, Recife, Pernambuco, Brazil; 2 Real Jardín Botánico, Department of Biodiversity and Conservation, Consejo Superior de Investigaciones Científicas, Madrid, Spain

**Keywords:** *Croton* section *Pedicellati*, Malpighiales, Neotropics, Phylogenetics, Taxonomy

## Abstract

A new species of *Croton,* microendemic of the dry forests of northeastern Brazil in the state of Pernambuco, is here described. Morphological and ITS molecular data provide evidence for placing *C. buiquensis* sp. nov. as part of *Croton* section *Pedicellati*. Specimens of *C. buiquensis* had been previously identified in herbaria as *C. pedicellatus*, but the former can be distinguished from the latter by the hispid indumentum of the leaf blade, the ovate shape of the petals of staminate flowers, pistillate flowers with 4-fid to multifid styles, and a crossbow-shape caruncle. Evidence from ecological niche modeling points to other areas in the state of Pernambuco, specifically the “Agreste” region, as likely locations for the occurrence of the new species, which could be the target of future botanical surveys. *Croton buiquensis* is here provisionally considered as Critically Endangered based on IUCN criteria. This is the first record of multifid styles (more than six stigmatic tips) in *C.* sect. *Pedicellati* and also the first species of the section endemic to the state of Pernambuco.

## Introduction

The current knowledge on the infrageneric classification of *Croton* L. in tropical America is synthesized in the work of Van Ee, Riina & Berry (2011). This classification placed all neotropical *Croton* species to section, guided by a molecular phylogenetic framework of representative taxa, and complemented by morphological and geographic evidence. Van Ee, Riina & Berry (2011) proposed 31 monophyletic sections corresponding to 31 well-supported clades. In this study, we propose and describe a new species that shows morphological affinities with *C.* sect. *Pedicellati*. This section comprises twenty species distributed disjunctly between Mexico and South America, with the majority of them occurring in central-western Brazil (11 spp.) (Van Ee & Berry, 2011; Van Ee, Riina & Berry, 2011). *Croton* sect. *Pedicellati* is currently described as monoecious, with alternate, petiolate, ovate-lanceolate, eglandular leaves, the presence of stellate to lepidote trichomes, short terminal inflorescences, pedicellate pistillate flowers with recurved pedicels, and bifid styles (Van Ee & Berry, 2011; Van Ee, Riina & Berry, 2011). All species in the section are found in scrubby tropical deciduous forests (Van Ee, Riina & Berry, 2011), which are patchy distributed across the neotropics, including those in dry inter-Andean valleys (DRYFLOR et al., 2016).

Most species of *C.* sect*. Pedicellati* occurring in Brazil are endemic to this country (Van Ee & Berry, 2011; Caruzo et al., 2020). Among them, six are endemic to the Cerrado (Brazilian savanna): *Croton angustifrons* Müll.Arg., *C. burchellii* Müll.Arg., *C. corchoropsis* Baill., *C. eriocladoides* Müll.Arg., *C. eriocladus* Müll.Arg., and *C. horminum* Baill.; two are endemic to the Caatinga: *C. catinganus* Müll.Arg. and *C. tridentatus* Mart. ex Müll.Arg.; and two to the Atlantic Forest: *C. dracunculoides* Baill. and *C. tenuicaulis* B.W.van Ee & P.E.Berry. Some species have very restricted distributions: *C. angustifrons*, recorded only in the state of Minas Gerais; *C. eriocladoides*, restricted to Goiás; and *C. tenuicaulis*, known exclusively from the state of Rio de Janeiro (Van Ee & Berry, 2011; Caruzo et al., 2020). These biogeographic patterns highlight the high degree of endemism of the section in Brazil and its importance in conservation.

As part of systematic studies on *C.* sect*. Pedicellati*, we encountered several specimens that morphologically did not correspond to any known *Croton* species. *Croton buiquensis* sp. nov. is only known from the state of Pernambuco, in the municipality of Buíque, with the first record dating back to 1970 (*Xavier-Filho & Alves*, *s.n.*). Like *C. buiquensis,* numerous new species with restricted distributions have been described in *Croton* with only one or few collection records (*e.g.*, Pereira, Riina & Caruzo, 2017; Farias, Medeiros & Riina, 2019; Sodré & Silva, 2022; Sodré et al., 2023; Tagane, Souvannakhoummane & Souldeth, 2022).

Here we describe *C. buiquensis* as a new species of *C.* sect. *Pedicellati* using evidence from molecular phylogenetics, morphology, ecology, and geographic distribution. The new species represents one of the three Caatinga-endemic species in its section, along with *C. catinganus* and *C. tridentatus* (Van Ee & Berry, 2011); and it is the first species of this group or clade endemic to the state of Pernambuco. We also estimate the species’ potential distribution using a niche modeling approach and provide a preliminary conservation status assessment based on all the data available.

## Materials and Methods

### Data collection and species description

The description of *C. buiquensis* was based on the analyses of collections from the herbaria (acronyms according to (Thiers, 2024), continuously updated): ALCB, BHCB, CEPEC, HUEFS, IPA, PEUFR, RB, SPF, UB and UFP and HST (not indexed). We followed the general terminology for shapes and types of indumentum of Radford, Dickison & Massey (1974). For trichomes terminology, we followed the classification proposed by Pinto-Silva et al. (2023).

The electronic version of this article in Portable Document Format (PDF) will represent a published work according to the International Code of Nomenclature for algae, fungi, and plants (ICN), and hence the new names contained in the electronic version are effectively published under that Code from the electronic edition alone. In addition, the new name contained in this work which has been issued with identifiers by IPNI will eventually be made available to the Global Names Index. The IPNI LSIDs can be resolved and the associated information viewed through any standard web browser by appending the LSID contained in this publication to the prefix “http://ipni.org/”. The online version of this work is archived and available from the following digital repositories: PeerJ, PubMed Central SCIE, and CLOCKSS.

### DNA extraction, amplification, and sequencing

The DNA used in the phylogenetic analyses was extracted from dried leaf tissue of herbarium specimens using the CTAB method (Doyle, 1991). We extracted DNA from the following specimens of the putative new species: *Laurenio A, 2013* (PEUFR); *Laurenio A, 376* (PEUFR); *Sales M. F., 1060* (PEUFR); *Carneiro-Torres D.,* 954 (HUEFS). After quantification (Qubit™ dsDNA BR Standard (Invitrogen)), the DNA samples were diluted (1:20) to perform PCR reactions to obtain the nuclear ITS genetic region. PCR amplifications were carried out in 25 µL reactions (12 µL MyTaq Red Mix (Bioline), 10 µL H2O, 1 µL of each primer (forward and reverse), and 1 µL DNA). The amplification primers were those used in recent studies in *Croton* and other Euphorbiaceae lineages (Silva et al., 2020; Külkamp et al., 2023). PCR products were purified with ExoSap PCR Purification and sent for sequencing at MACROGEN (Macrogen, Madrid, Spain) using the same PCR amplification primers. The nucleotide sequences were assembled and edited in UGENE 33.0 (Okonechnikov et al., 2012) and automatically aligned using the online version of MAFFT (Katoh, Rozewicki & Yamada, 2019) with default parameters. Manual adjustments were made to the alignment matrix in UGENE 33.0, applying the similarity criterion (Simmons, 2004).

### Phylogenetic analyses

The newly generated ITS sequence of *C*. *buiquensis*, along with a new sequence of *C. breedlovei* (another species in *C.* sect. *Pedicellati* from Mexico), were aligned together with a selection of publicly available ITS sequences of *Croton*. This selection included all sequenced species of *C*. sect. *Pedicellati* and its sister clade *C*. sect. *Lamprocroton* (Van Ee & Berry, 2011), and representatives of *Croton* sections: *Barhamia*, *Cleodora*, *Cuneati*, *Cupreati*, *Julocroton*, *Lasiogyne*, *Luetzelburgiorum*, and, as outgroup, *Adenophylli*, obtained from GenBank (https://www.ncbi.nlm.nih.gov/genbank/). In total, our taxon sampling included 31 ITS sequences representing 29 *Croton* species. Ten of these sequences belonged to *C*. sect. *pedicellati*, representing eight species, including the new taxon. A complete list of species, their systematic positioning, vouchers, and deposition herbaria is presented in File S1. Outgroups were selected based on previous molecular studies (*e.g.*, Van Ee & Berry, 2011; Silva et al., 2020; Masa-Iranzo et al., 2021). The ITS matrix was trimmed at both ends to minimize the effects of missing data due to unequal sequence sizes. The aligned matriz used in the analysis is provided along with the two newly generated sequences (File S2). Bayesian Inference were performed in MrBayes ver. 3.2.7 (Ronquist et al., 2012), applying the GTR+I+G model following Masa-Iranzo et al. (2021). Posterior probability (PP) values were obtained from two Markov Chain Monte Carlo (MCMC) analyses, each consisting of four chains for 10 million generations, with sampling every 1,000th generation. Tracer v1.7.2 (Rambaut, 2018) was used to check the convergence through Effective Sample Size value (ESS > 200). A 50% majority rule consensus tree was built after discarding the first 25% of the samples as burn-in. Phylogenetic analyses were hosted on the CIPRES Science Gateway (Miller, Pfeiffer & Schwartz, 2010). The software FigTree ver. 1.4.4 (Rambaut, 2018) was used to visualize and edit the phylogenetic tree. In addition to the Bayesian inference, we conducted a Maximum Likelihood analysis in IQ-TREE version 1.6.12 (Lam-Tung et al., 2015), using the same model above. Differences in genetic distance (Jukes & Cantor, 1969) between *C*. *buiquensis* and the other species of *C*. sect. *Pedicellati* were estimated in PAUP* v 4.0a (Swofford, 2002).

### Occurrence data and preliminary assessment of the species conservation status

Occurrence data for the species were compiled from information present in herbarium specimens, and distribution maps were generated using QGIS 3.8 software (https://qgis.org/). A preliminary assessment of the species conservation status was conducted based on IUCN Criteria B1 and B2 (IUCN & Petitions Committee, 2024), supplemented by the calculation of Extent of Occurrence (EOO) and Area of Occupancy (AOO), using the GeoCAT tool (Bachman et al., 2011).

### Ecological niche modeling

The occurrence data used for distribution map and niche modeling analysis (File S3) along with the Caatinga shapes (File S4) are provided as supplementary files. A set of 19 bioclimatic variables was obtained from the WorldClim version 1.4 database (Fick & Hijmans, 2017), with a spatial resolution of 30 s (using the limits of the Caatinga region of Brazil). To avoid multicollinearity issues and improve model robustness, a 70% correlation threshold was applied in the selection of environmental variables (Booth et al., 2014). After this analysis, seven variables were selected as predictors (Table S1).

The species’ potential distribution was modeled using the modleR package (Sánchez-Tapia et al., 2020). Cross-validation techniques were employed to evaluate model accuracy and robustness. The Maxent algorithm was used to estimate the potential distribution; this algorithm applies the maximum entropy method to model species distribution using presence data and environmental variables (Phillips et al., 2017). The use of Maxent is particularly suitable for species with few occurrence records, with five to 10 unique occurrence points (not spatially duplicated) being the minimum required to run the analyses (Phillips, Anderson & Schapire, 2006). It performs well with small sample sizes by relying on presence-only data and incorporating regularization to prevent overfitting (Wisz et al., 2008; Phillips, Anderson & Schapire, 2006; Hernandez et al., 2006). The model’s accuracy was assessed using the area under the ROC curve (AUC). A calibration area for each species was defined by creating a minimum convex polygon around all occurrences based on 100% of occurrence points, surrounded by a 1.5° buffer. The projection area was defined by the limits of the Caatinga region of Brazil, as it corresponds to the biome where the species occurs.

The environmental suitability area was calculated from the distribution models using the “cellStats”() function from the raster package (Hijmans, 2024) in R software (v. 4.4.0, R Core Team, 2023). A suitability threshold of 0.6 was applied to generate binary maps, considering cells with values above the threshold as suitable. From the suitability area indicated in the ecological niche modeling, the extent of the territory (km^2^) protected was calculated using the mask_thr_projs_mscn_b function from the ENMwizard package (Heming, Dambros & Gutiérrez, 2019). Data on protected areas were obtained from UNEP-WCMC and IUCN databases (UNEP-WCMC & IUCN , 2023). All analyses were conducted in R software (v. 4.4.0, R Core Team, 2024).

## Results

### Taxonomy and morphological affinities

Our specimens survey showed that *C. buiquensis* had often been misidentified in herbarium collections as *C. pedicellatus* due to some morphological similarities like its habit and leaf blade form. The morphological analysis of the available specimens indicated that the new species can be distinguished from *C. pedicellatus* by the indumentum of the leaf blade, the shape of the staminate petal and the styles. Overall, *C. buiquensis* exhibits morphological traits that are diagnostic for *Croton* sect. *Pedicellati*, such as herbaceous to shrubby habit, entire leaves, acropetiolar nectary glands absence, indument with stellate to sublepidote trichomes, terminal inflorescence. However, it can be distinguished from the rest of the species in the section by its multifid styles (*vs.* bifid styles).

### Phylogenetic placement and relationships

Specimens of *C. buiquensis* were extremely difficult for PCR amplification and sequencing, even when doing the amplification of the ITS region with internal primers (*i.e.,* sequencing it in two parts). Of the five specimens extracted for DNA only one (*Carneiro-Torres 954*; File S1) yielded a complete ITS sequence after several attempts using internal primers.

**Figure 1:**
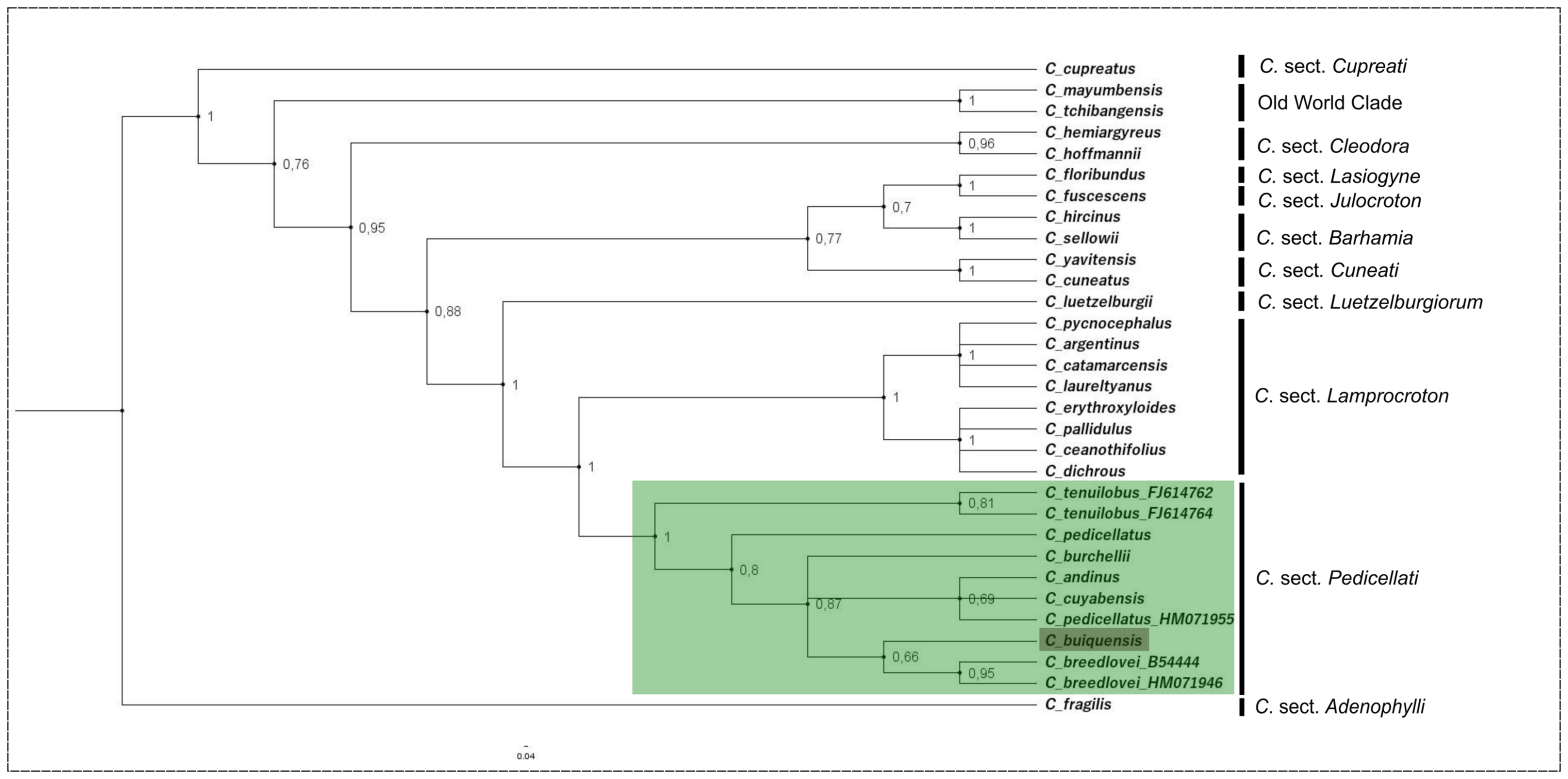
*Croton buiquensis* sp. nov. in a phylogenetic context. Bayesian phylogram based on ITS sequences of the selected *Croton* species including *C. buiquensis* and representatives of *C*. section *Pedicellati* and other sections in the genus. Numbers at nodes indicate Bayesian posterior probability values (PP).

The results of the phylogenetic analyses conducted using Bayesian inference and maximum likelihood were practically identical in terms of topology and clade support. The Bayesian tree (Fig. 1) is used to describe the phylogenetic structure and relevant relationships. The representative species from the *Croton* sections sampled for our analysis formed monophyletic groups with high support value (PP = 0.95–1; Fig. 1). The species of *C.* sect*. Pedicellati* were also recovered in a clade (PP = 1), and sister to the *C.* sect. *Lamprocroton* clade (PP = 1).*Croton buiquensis* was recovered within the *C.* sect. *Pedicellati* clade, forming a moderately supported subclade together with two accessions of *C. breedlovei* (PP = 0.66; Fig. 1). The genetic distance of *C. buiquensis* from other species from *C.* sect. *Pedicellati* were low, especially when compared with the two accessions of *C. breedlovei* (represented by two different DNA isolates from the same voucher), its most closely related species given the available taxa with sequences (distance values 0.00982; Fig. S1).

### Niche modeling

**Figure 2:**
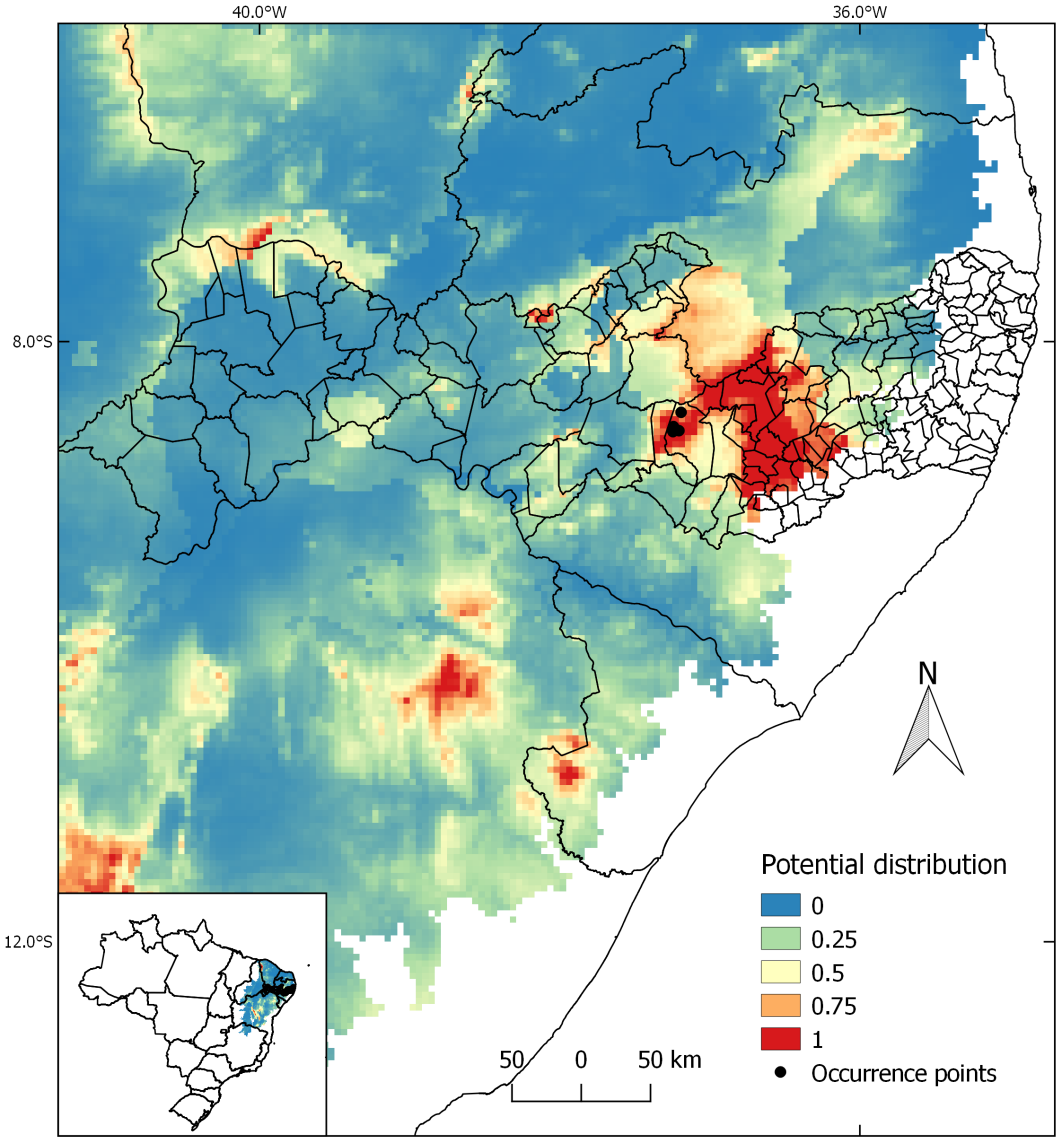
Niche modeling of *Croton buiquensis* sp. nov. Blue color represents regions with low climatic suitability and orange and red colors represent regions with high climatic suitability. The colored background represents the area of the Caatinga domain (see Methods).

Seven georeferenced points, not spatially duplicated, of *C. buiquensis* were confirmed and used to produce the models. The AUC value for niche modeling analysis was satisfactory (= 0.98). The generated models identified potential distribution areas of the species in a considerable portion of the “Agreste Pernambucano” mesoregion, mainly in the southern part of the state (Fig. 2), where the municipality of Buíque is located. The “Agreste” region represents a transitional zone between the “Zona da Mata”, characterized by a humid climate, and the “Sertão”, where the climate is semi-arid. It exhibits intermediate environmental conditions, with predominance of caatinga vegetation interspersed with patches of more humid vegetation at higher elevations, known as montane forests or “brejos de altitude.” The models also indicated other areas in the Northeastern region of Brazil, in the states of Bahia and Sergipe, with high potential for the species’ occurrence (Fig. 2). The occurrence points of *C. buiquensis* plotted on the map were located in the areas of highest probability of occurrence. The total area of suitability indicated by the models was 826,533.7 km^2^, of which only 43,995.86 km^2^ are protected by some conservation unit.

### Distribution and preliminary conservation status assessment

**Figure 3:**
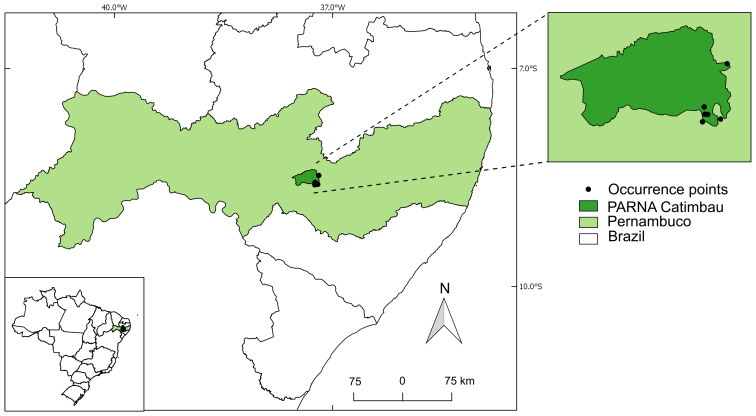
Geographic distribution map of *Croton buiquensis* sp. nov.

*Croton buiquensis* has been only recorded in the municipality of Buíque, in the state of Pernambuco, northeastern Brazil, within the Catimbau National Park (a conservation unit with full protection, according to Brazilian legislation) (Fig. 3). Considering the extent of occurrence (EOO) of 87.682 km^2^ and its area of occupancy (AOO) of 8 km^2^, the species is classified as Critically Endangered (CR). Information about the health of the populations, including the number of mature individuals, is currently lacking.

### Taxonomic treatment

**Figure 4:**
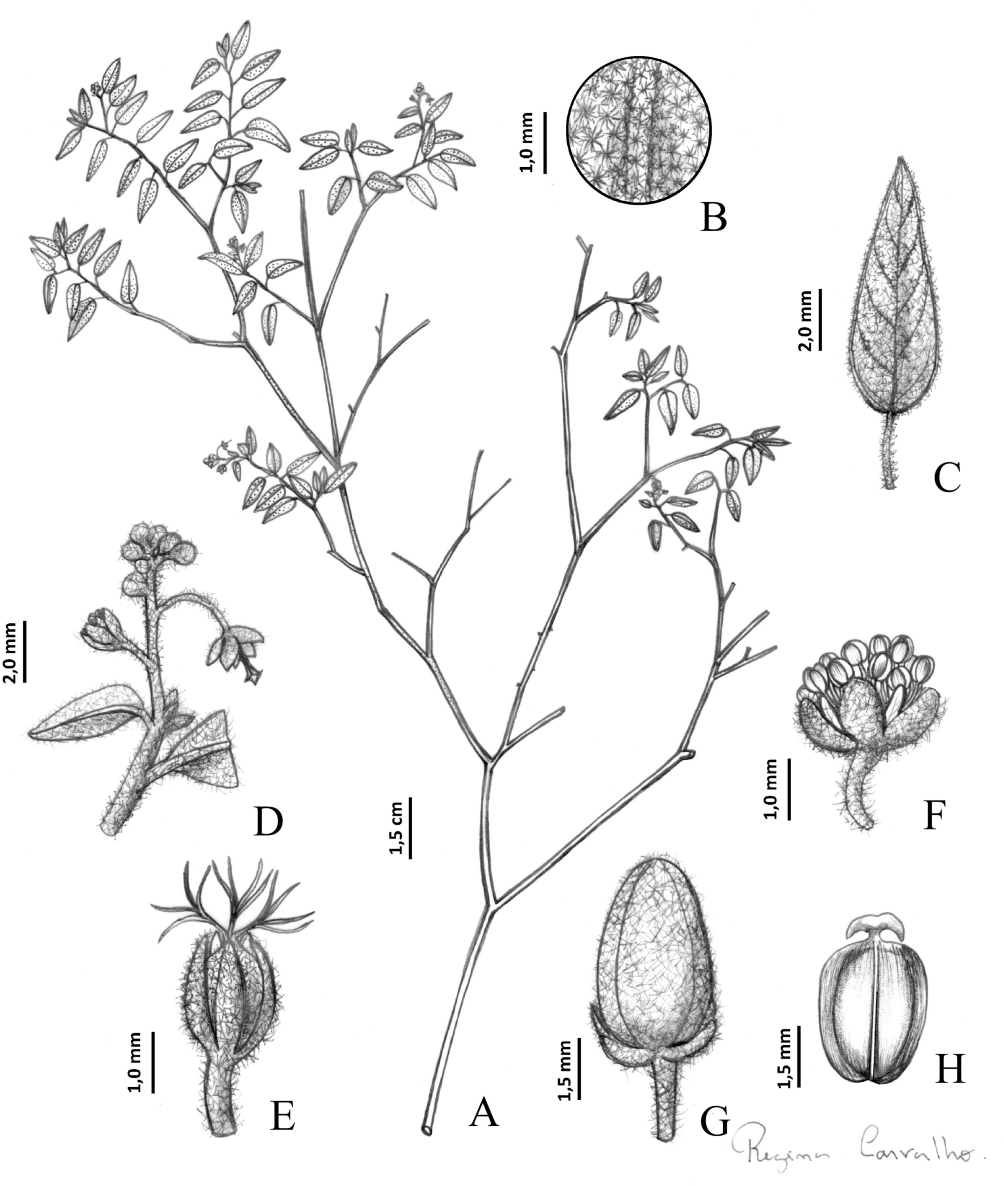
Main morphological features distinguishing * Croton buiquensis* sp. nov. *Croton buiquensis* sp. nov. (A) Flowering branch. (B) Indumentum on the abaxial side of the leaf blade. (C) Detail of a leaf. (D) Detail of an inflorescence. (E) Pistillate flower. (F) Staminate flower. (G) Capsule. (H) Seed. A from *Laurênio A. 123* (PEUFR); (B, C) and (D) from *Cano O. 791* (IPA); (E, F, G) and (H) from *Fontana AP 10096* (PEUFR).

***Croton buiquensis*** J.C.P. Oliveira **sp. nov.**—TYPE: Brazil, Pernambuco, Buíque, Parque Nacional da Serra do Catimbau, subida para Serra das Torres; 8°33′60″S, 37°14′28″W; 28 Jun 2007, fl., *Carneiro-Torres D*. *954*; holotype: HUEFS (HUEFS000106140!), isotypes: HUESB-JQ (HUESB2980), SP (SP0396823!) (Fig. 4).

### Etymology

The specific epithet of the new species refers to the municipality of Buíque in the state of Pernambuco where all specimens of *C. buiquensis* have been recorded to date. *Buíque* is a word of Tupi origin, the main indigenous language spoken in Brazil until the mid-18th century, and it means *place of snakes*.

### Diagnosis

Besides its geographic range, *C. buiquensis* can be distinguished from other species in *C*. sect. *Pedicellati* by its 4-fid to multifid styles, the presence of sublepidote trichomes on branches and leaf blades, and the crossbow-shape caruncle.

### Description

Herbs to subshrubs, monoecious, approximately 50 cm tall. Branches ferruginous to grayish, indumentum hispid to glabrous, sometimes tomentose, with stellate to sublepidote trichomes; stipules 0.8–1.0 mm long, ovate, hispid indumentum, stellate trichomes. Petiole 0.4–0.7 cm long, hispid, stellate to porrect-stellate trichomes (porrect ray measuring 60–100 µm), eglandular. Leaves alternate, chartaceous, leaf blades ovate to ovate-lanceolate, 1.1–1.6 × 0.4–0.7 cm, venation eucamptodromous, apex acute, base rounded, adaxial surface hispid, with densely grouped stellate trichomes, abaxial surface hispid, stellate trichomes, sometimes ferruginous, margin entire. Inflorescence a thyrse, 0.4–1.7 cm long, bracts ovate, approximately 1.0 mm long, hispid to pubescent, stellate trichomes, pistillate flowers located at the base of the axis and staminate flowers on the distal portion, sometimes flowers are very close to one another due to the short length of the inflorescence axis. Staminate flowers ca. 1.4 × 1.7 mm, pedicel 2.0–2.1 mm long, hispid to pubescent, stellate trichomes; sepals 5, ca. 1.0 × 1.1 mm, ovate to elliptical, hispid to pubescent, stellate trichomes; petals 5, ca. 0.3 × 1.0–1.1 mm, ovate to ovate-lanceolate, glabrous, margin entire, sometimes with long simple trichomes, appearing ciliate; stamens 10, 1.1–1.2 mm long. Pistillate flowers ca. 2.0 × 2.8 mm, pedicels 0.3–8.0 mm long; sepals 5, ovate, 2.0–2. 1 × 0.7 mm, free from one another, apex acute, margin entire, hispid to pubescent indumentum, stellate trichomes; styles 4-fid to multifid, glabrous to pubescent, stellate, porrect trichomes; petals absent; ovary globose, approximately 1.1 mm in diameter, hispid to pubescent, with stellate trichomes. Capsules ellipsoid, ca. 0.3 × 0.5 cm, hispid indumentum, stellate, porrect trichomes; fruiting pedicel 4.0–7.0 mm long; columella 2.0–3.0 mm long. Seeds rounded, slightly rough, approximately 3.0–4.0 × 3.0 mm, caruncle crossbow-shape.

### Paratypes

**BRAZIL. Pernambuco:** Buíque, Parque Nacional do Catimbau, Chapada São José, 8°37′23″S, 37°09′21″W, 14.IX.2011, fl., *Costa A. C. G. et al. 46* (IPA); Chapada de São José; 8°37′23″S 37°09′21″W, 03.IV.2000, *Sales M. 1060* (JPB, PEUFR, UFP); Estrada Buíque Catimbau, 37°09′15″W, *Laurênio A. 123* (K, NY, PEUFR); Parna Catimbau; 8°35′37″S 37°12′18″W, 01.VII.2015, *Felix L.P. 15663* (CSTR, EAN); Parque Nacional do Catimbau, Serrinha, 8°37′23″S 37°09′21″W, 13.I.2011, *Costa A. C. G. et al. 12* (IPA); Sobrado, Serra das Andorinhas, 8°28′17″S 37°11′26″W, 30.VIII.2018, *Fontana A. P. 10096* (HUEFS); Serra do Catimbau, 8°37′23″S 37°09′21″W, 15.X.1970, *Xavier-Filho L. & Alves J. L. H. s.n.* (UFP); Vale do Catimbau, 8°37′23″S 37°09′21″W, *Costa Filho L. O. s.n.* (UFP); Vale do Catimbau, Paraiso Tropical, alto do lajeiro, 8°34′58″S 37°14′23″W, 18.VII.2007, *Cano O. et al. 791* (IPA, HUEFS); Vale do Catimbau, 8°37′23″S, 37°09′21″W, fl., *Gomes A. P. S. et al. 509* (IPA); Vale do Catimbau, Serra das Torres, Savana Estépica/caatinga, 8S 35′58″, 37°14′41″W, 866 m.a.s.l., 29.XI.2016, fl., *Oliveira M. 6509* (HUEFS, PISF, RB).

### Habitat and ecology

*Croton buiquensis* is only known from the municipality of Buíque, in the state of Pernambuco, northeastern Brazil, within the Catimbau National Park. It grows in hilly areas of steppe savanna with dense shrub vegetation and/or shrub-arboreal vegetation, as well as on rocky outcrops, at elevations ranging from 780 to 900 m. The collected specimens were found flowering between April and October.

### Notes

**Figure 5:**
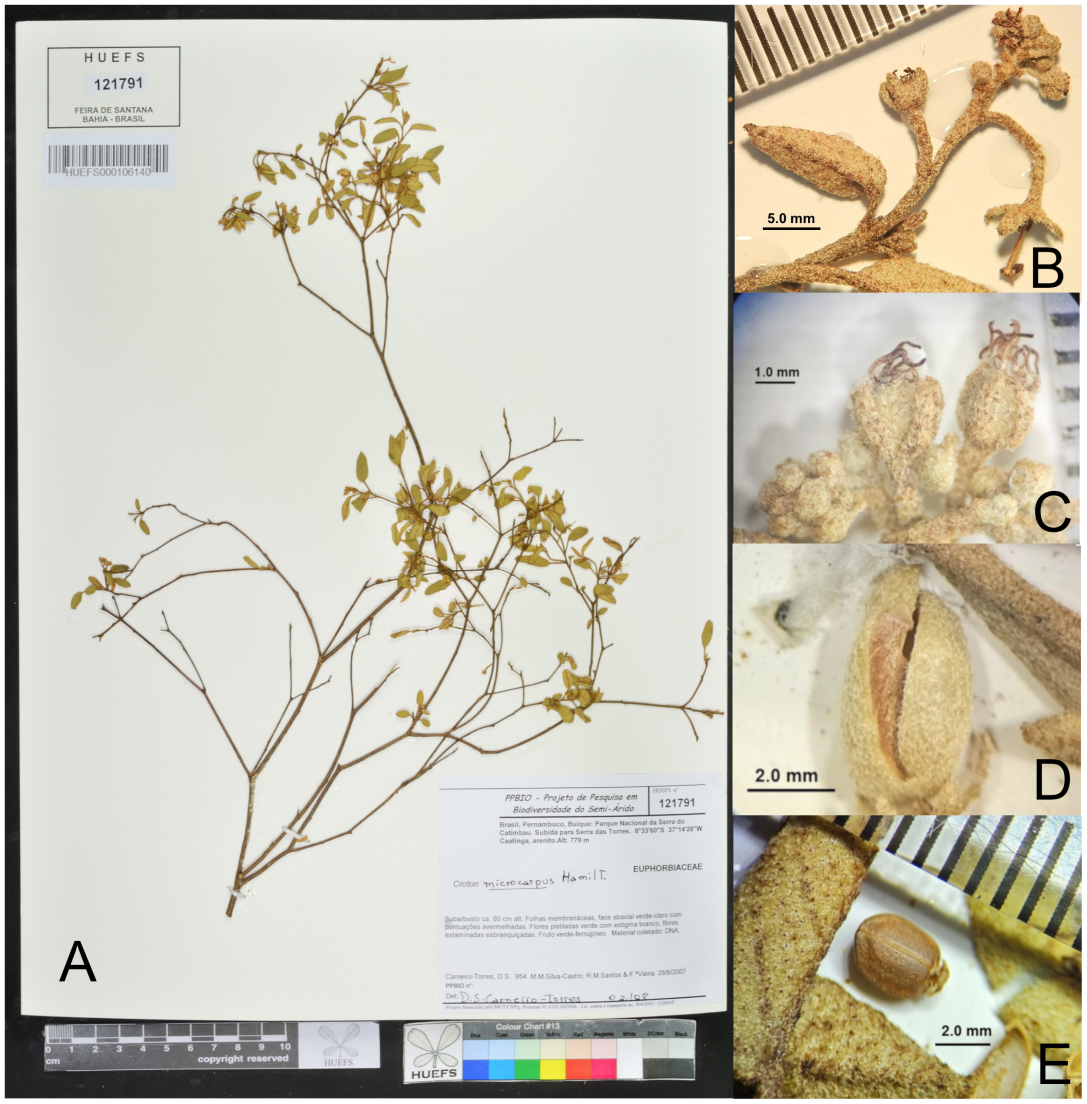
Herbarium images of *Croton buiquensis* sp. nov. *Croton buiquensis* sp. nov. (A) Image of the type at HUEFS. (B) Inflorescence, showing pistillate flowers and staminate cymules. (C) Pistillate flower at anthesis showing the pedicel 4-multifid styles and oval to elliptical sepals. (D) Opened capsule stage shown from the side. (E) Seed shown from the front side, and the crossbow shaped caruncle; abaxial leaf surface on the left showing the ferrugineous trichomes. (A) from *Carneiro-Torres 954* (HUEFS); (B) and (E) from *Cano O. 791*; (C) and (D) *Fontana AP 10096* (PEUFR).

The crossbow-shape caruncle of *C. buiquensis* appears to be unique within *Croton*. It is characterized by an arched and elongated structure, resembling a crossbow, with lateral projections and a short, central stalk attached to the apical part of the seed (Figs. 4H and 5E). The crossbow-shape differs from the known caruncle shapes present in most *Croton* species (*e.g.*, reniform, trapezoidal, oblong, elliptic, winged) (*e.g.*, Riina et al., 2010; Secco et al., 2012; Anton et al., 2024; Pereira et al., 2025).

## Discussion

Overall, the topology of our phylogenetic analysis at the genus level (backbone) and within section *Pedicelati* is congruent with previous ITS phylogenetic analyses of *Croton* (Van Ee & Berry, 2011; Van Ee, Riina & Berry, 2011; Arévalo et al., 2017). The membership of *C. buiquensis* to *C*. sect. *Pedicellati* is supported by morphological traits shared with other species in this clade, including herbaceous to shrubby habit, entire leaves, absence of acropetiolar nectary glands, stellate trichomes on both leaf sides and ovate pistillate sepals (Van Ee & Berry, 2011). However, as indicated in the diagnosis above, there are three key morphological characters differentiating *C*. *buiquensis* from the other species in the section (Table 1). The new species represents the first record of 4-fid and multifid styles in *C.* sect. *Pedicellati*, thereby justifying an adjustment to the description of the section to reflect the current variation in style branching: bifid, 4-fid or multifid styles. Tetrafid (multifid) styles are also present in *C.* sect*. Lamprocroton*, the sister section to *C.* sect*. Pedicellati* and other sections of *Croton* (Lima de & Pirani, 2008; Van Ee & Berry, 2011).

**Table 1:** Main morphological differences between *Croton buiquensis* sp. nov. and some of the most similar species of the *C.* sect. *Pedicellati*.

Feature	*C. buiquensis* sp. nov.	*C. pedicellatus*	*C. breedlovei*	*C. catinganus*
Abaxial leaf surface (indumentum)	Hispid	Hirsute-pubescent to pubescent	Lepidote	Woolly a pubescent
Styles	4-fid to multifid	Bifid	Bifid	Bifid
Branches (indumentum)	Hispid to glabrous, sometimes tomentose; stellate and sublepidote trichomes	Hirsute-hispid to glabrescent; stellate and stellate porrect trichomes	Lepidote; stellate and lepidote trichomes	Hirsute to glabrescent; stellate trichomes
Caruncle shape	Crossbow shape	Collar-like or irregularly mantle-shaped	Lunate	reniform
Geographic distribution	Brazil (Pernambuco)	Brazil (widespread)	Mexico (Chiapas region)	Brazil (Bahia, Paiuí)

In general *Croton* species are relatively easy to sequence for the ITS region as demonstrated for the numerous phylogenetic publications using this genetic marker (*e.g.*, Berry et al., 2005; Van Ee, Riina & Berry, 2011; Rossine et al., 2025). However, *C. buiquensis* appears to be an exception. It took us many tries in the molecular lab using material from five different herbarium specimens until we finally obtained one ITS sequence for the phylogenetic analysis. It is possible that the chemistry of this species includes one or more compounds that interfere with the PCR amplification process. *Croton* and Euphorbiaceae in general are well known for their highly diverse chemistry (Salatino, Salatino & Negri, 2007; Lopes et al., 2012; Coelho et al., 2016; Xu, Liu & Liang, 2018; Guerra-Júnior et al., 2022), including numerous secondary metabolites, so it is not surprising that PCR inhibitors could be present in some species.

Specimens of *C. buiquensis* had been misidentified in herbaria as *C. pedicellatus* this confusion is probably due to the high similarity in some morphological features between the two species. However, the new species can be distinguished from *C. pedicellatus* by the indumentum of the leaf blade (hispid *vs.* hirsute-pubescent to pubescent in *C. pedicellatus*), the shape of the staminate petal (ovate *vs.* oblong), and the style (4-fid to multifid *vs.* bifid). The distinction of the type of leaf blade indumentum is due to differences in the length of the central (porrect) ray of the trichomes.

The phylogenetic analysis (Fig. 1) as well as the Jukes-Cantor analysis (Fig. S1), shows that *C. buiquensis* is genetically close to *C. breedlovei*, a species endemic to the Chiapas region in Mexico (Van Ee & Berry, 2011). Despite the phylogenetic proximity, the two species can be distinguished by their geographical distribution as well as by several morphological characters (see Table 1). Disjunct distribution patterns have also been reported in other *Croton* lineages, which show fragmented geographic ranges across Neotropics (*e.g.*, Riina et al., 2018; Riina et al., 2021). This fragmented geographic pattern is also shown below the species level with species showing disjunct populations at continental or regional scales (*e.g.*, Caruzo et al., 2016; Rossine et al., 2025).

Considering that *C. buiquensis* occurs within a permanent preservation area (Catimbau National Park), one might assume that the species is not as exposed to threats. However, areas with rupestrian field vegetation are quite sensitive to climate change, and recent future projections for these environments show considerable reduction in size, even in protected areas (Fernandes et al., 2018). Despite being a permanent conservation unit, Catimbau National Park is not immune to environmental degradation processes due to anthropogenic actions such as ecotourism and housing (Vranckx & Castilho, 2010; Silva & Maia, 2011; Azevedo & Bernard, 2015). Protected areas are also impacted by the establishment of invasive exotic species (Silva & Fabricante, 2019), which subject endemic native species to a high degree of threat. For these reasons, *C. buiquensis* is here considered as critically endangered pending formal evaluation by the IUCN.

The results of the ecological niche modeling point to other areas in the “Agreste Pernambucano” region as likely locations for the species’ occurrence. These areas have similar climatic conditions associated with sub-humid and semi-arid climates, over pediplains or mountain ranges (Albuquerque et al., 2019). However, no records of this species have been found in these areas up until now. Thus, the species is for now considered restricted to the municipality of Buíque, being the first species of *C*. sect. *Pedicellati* endemic to the state of Pernambuco, but we believe that further collection efforts in these areas are needed. The models also identified other areas in the states of Bahia and Sergipe as having high potential for the species’ occurrence. However, models can overestimate species distribution, as the environmental conditions predicted for an ecological niche may be present in several locations within a relatively large geographic area (Urbina & Loyola, 2008; Jetz, Sekercioglu & Watson, 2008; Suarez-Contento et al., 2024). The existence of potential areas outside the current distribution of the species does not guarantee that it can effectively colonize these regions. Factors such as limitation in dispersal capacity, species competition or biological barriers can prevent geographic range expansion (Mendes et al., 2020; Velazco et al., 2020).

## Conclusions

The description of *C. buiquensis* as a new endemic species from Northeast Brazil underscores the importance of continuous efforts in documenting the flora in highly diverse and often underexplored regions of the Neotropics. This study not only positions the species within *C.* sect*. Pedicellati* but also provides essential information about its ecology and potential distribution based on niche models. The new species also expands the morphological concept of *C*. sect. *Pedicellati*, which should now include bifid and multifid styles as well as sublepidote trichomes. In addition, this is the first time that a crossbow-shape caruncle is described in *Croton*. The restricted geographic range of *C. buiquensis*, combined with anthropogenic pressure on ecosystems in the Brazilian semi-arid region, including legally established conservation areas, reinforces its threatened status and the need for additional conservation strategies.

## Supplemental Information

10.7717/peerj.20718/supp-1 Supplemental Information 1 Supplementary Files

10.7717/peerj.20718/supp-2 Supplemental Information 2 Sequences of ITS (fasta format) used in the study Previously published and newly generated sequences.

10.7717/peerj.20718/supp-3 Supplemental Information 3 Occurrence points (coordinates) of Croton buiquensis sp. nov

10.7717/peerj.20718/supp-4 Supplemental Information 4 Shapefiles of the Brazilian Caatinga region used in the niche modeling analysis of Croton buiquensis

## Additional Information and Declarations

### DNA Deposition

The following information was supplied regarding the deposition of DNA sequences: The two new sequences are available at GenBank: C. breedlovei van Ee & P. E. Berry; Mexico, Chiapas; Breedlove & Davidse 54444 (MICH); HM071946; PX400567 *. C. buiquensis sp. nov., Brazil, Pernambuco, Buíque, Carneiro 954 (HUEFS); PX400567 *. The already published sequences are available at GenBank: C. andinus Müll.Arg.; Argentina, Catamarca; van Ee et al. 657 (US); FJ614761. C. argentinus Müll.Arg.; Argentina, Córdoba; van Ee et al. 644 (US); HM071943. C. burchellii Müll.Arg.; Brazil, Goias; Anderson et al. 6827 (MO); HM071947. C. catamarcensis Ahumada; Argentina, Córdoba; Van Ee & al. 653 (US); HM071949. C. ceanothifolius Baill.; Brazil, Minas Gerais; Lima 352 (WIS); HM071950. C. corchoropsis Baill.; Brazil, Piauí; Carneiro-Torres 800 (SP); HM071955. C. cuneatus Klotzsch; Peru; Riina 1491 (MICH); EU478005. C. cupreatus Croizat; Ecuador, Pichincha; Riina 1408 (WIS); EU586919. C. cuyabensis Pilger; Brazil, Goias; Allem 3065 (MICH); HM071951. C. dichrous Müll.Arg.; Brazil, São Paulo; Berry 7691 (MICH); HM071952. C. erythroxyloides Baill.; Brazil, São Paulo; Caruzo et al. 74 (SP); EU586938. C. floribundus Spreng.; Brazil, São Paulo; Santos; 3 (SP); OR622587. C. fragilis Kunth; Venezuela, Sucre; Riina 1295 (VEN); MW263128. C. fuscescens Spreng.; Brazil, Ceará; Sousa; 4 (HUEFS); OR622588. C. hemiargyreus Müll.Arg.; Brazil, Minas Gerais; Caruzo 114 (SP); HM044793. C. hircinus Vent.; Venezuela, Distrito Federal; Riina; 1291; (VEN); EU477889. C. hoffmannii Müll.Arg.; Costa Rica, Cartago; Van Ee 598 (WIS); EF421773. C. laureltyanus Ahumada; Argentina, Corrientes; Belgrano et al. 281 (SI); HM071953. C. luetzelburgii Pax & K.Hoffm.; Brazil, Bahia; Conceição; 1457; (HUEFS); HM564087. C. mayumbensis J.Léonard; Gabon; Bissiengou 704 (WAG); MW263163. C. pallidulus Baill.; Brazil, São Paulo; Caruzo et al. 31 (SP); EU586939. C. pedicellatus Kunth; Colombia, Cundinamarca; Plowman 3766 (GH); FJ614766. C. pycnocephalus Baill.; Brazil, Rio Grande do Sul; Rossato et al. 13237 (MO); FJ614708. C. sellowii Baill.; Brazil, Bahia; Van Ee 498 (WIS); HM564095. C. tchibangensis Pellegr., Gabon, Nyanga, Valkenburg 2668 (WAG); MW263164. C. tenuilobus S.Watson; Mexico, Jalisco; Webster & Lynch 17169 (GH); FJ614764. C. tenuilobus; Mexico, Sinaloa; Gentry 5532 (GH); FJ614762. C. yavitensis Croizat; Bolivia, Beni; Beck 5710 (LPB); EU586918.

### Data Availability

The following information was supplied regarding data availability: The data is available in the Supplemental Files. The sequences are accessible *via* GenBank accession numbers PX400567–PX400568.

### New Species Registration

The following information was supplied regarding the registration of a newly described species: *Croton buiquensis* J.C.P. Oliveira.
