# Supplementary material for: Croton buiquensis (Euphorbiaceae), a new species from northeastern Brazil, its phylogenetic placement, and niche modeling
Source: PeerJ. 2026 Feb 6;14:e20718. doi: 10.7717/peerj.20718 (PMC12884962; doi:10.7717/peerj.20718)
Supplement: Supplemental Information 1 [file peerj-14-20718-s001.docx]

**Supplementary File 1**

**Taxa, localities, vouchers, and GenBank accession numbers for all sequences of the nuclear ITS region analyzed in this study.** The accession number of the two newly generated sequences are marked with an asterisk (*).

***C. andinus*** Müll.Arg.; Argentina, Catamarca; van Ee et al. 657 (US); FJ614761. ***C. argentinus*** Müll.Arg.; Argentina, Córdoba; van Ee et al. 644 (US); HM071943. ***C. breedlovei*** van Ee & P. E. Berry; Mexico, Chiapas; Breedlove & Davidse 54444 (MICH); HM071946; PX400567*. ***C. buiquensis*** sp. nov., Brazil, Pernambuco, Buíque, Carneiro 954 (HUEFS); PX400567*. ***C.*** ***burchellii*** Müll.Arg.; Brazil, Goias; Anderson et al. 6827 (MO); HM071947. ***C.*** ***catamarcensis*** Ahumada; Argentina, Córdoba; Van Ee & al. 653 (US); HM071949. ***C.*** ***ceanothifolius*** Baill.; Brazil, Minas Gerais; Lima 352 (WIS); HM071950. ***C.*** ***corchoropsis*** Baill.; Brazil, Piauí; Carneiro-Torres 800 (SP); HM071955. ***C.*** ***cuneatus*** Klotzsch; Peru; Riina 1491 (MICH); EU478005. ***C. cupreatus*** Croizat; Ecuador, Pichincha; Riina 1408 (WIS); EU586919. ***C.*** ***cuyabensis*** Pilger; Brazil, Goias; Allem 3065 (MICH); HM071951. ***C.*** ***dichrous*** Müll.Arg.; Brazil, São Paulo; Berry 7691 (MICH); HM071952. ***C.*** ***erythroxyloides*** Baill.; Brazil, São Paulo; Caruzo et al. 74 (SP); EU586938. ***C.*** ***floribundus*** Spreng.; Brazil, São Paulo; Santos 3 (SP); OR622587. ***C. fragilis*** Kunth; Venezuela, Sucre; Riina 1295 (VEN); MW263128. ***C. fuscescens*** Spreng.; Brazil, Ceará; Sousa 4 (HUEFS); OR622588.  ***C. hemiargyreus*** Müll.Arg.; Brazil, Minas Gerais; Caruzo 114 (SP); HM044793. ***C. hircinus*** Vent.; Venezuela, Distrito Federal; Riina 1291 (VEN); EU477889.  ***C. hoffmannii*** Müll.Arg.; Costa Rica, Cartago; Van Ee 598 (WIS); EF421773. ***C.*** ***laureltyanus*** Ahumada; Argentina, Corrientes; Belgrano et al. 281 (SI); HM071953. ***C.*** ***luetzelburgii*** Pax & K.Hoffm.; Brazil, Bahia; Conceição 1457  (HUEFS); HM564087. ***C. mayumbensis*** J.Léonard; Gabon; Bissiengou 704 (WAG); MW263163. ***C.*** ***pallidulus*** Baill.; Brazil, São Paulo; Caruzo et al. 31 (SP); EU586939. ***C.*** ***pedicellatus*** Kunth; Colombia, Cundinamarca; Plowman 3766 (GH); FJ614766. ***C.*** ***pycnocephalus*** Baill.; Brazil, Rio Grande do Sul; Rossato et al. 13237 (MO); FJ614708. ***C.*** ***sellowii*** Baill.; Brazil, Bahia; Van Ee 498 (WIS); HM564095. ***C. tchibangensis*** Pellegr., Gabon, Nyanga, Valkenburg 2668 (WAG); MW263164. ***C.*** ***tenuilobus*** S.Watson; Mexico, Jalisco; Webster & Lynch 17169 (GH); FJ614764. ***C.*** ***tenuilobus***; Mexico, Sinaloa; Gentry 5532 (GH); FJ614762. ***C.*** ***yavitensis*** Croizat; Bolivia, Beni; Beck 5710 (LPB); EU586918.

**Table S1**. **Variables selected as predictors for niche modeling analysis.**

| **Code** | **Bioclimatic variables** |
| --- | --- |
| BIO_3 | Isothermality |
| BIO_7 | Temperature Annual Range |
| BIO_10 | Mean Temperature of Warmest Quarter |
| BIO_12 | Annual Precipitation |
| BIO_14 | Precipitation of Driest Month |
| BIO_18 | Precipitation of Warmest Quarter |
| BIO_19 | Precipitation of Coldest Quarter |

**
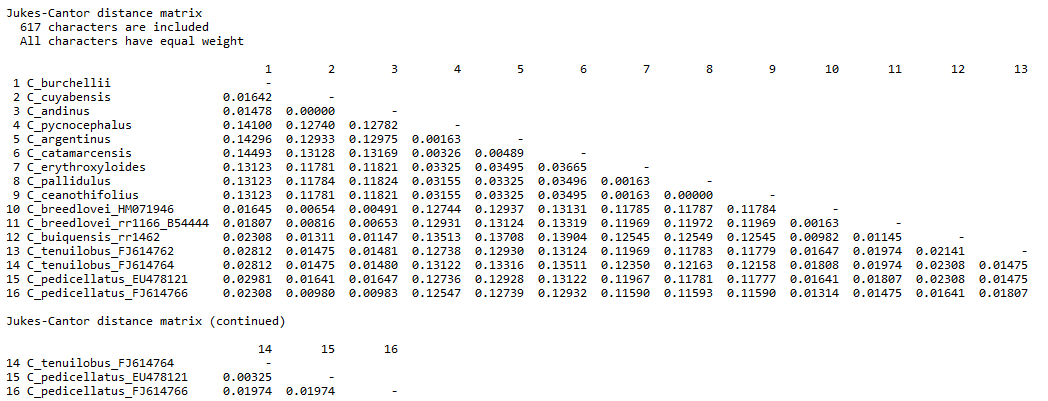
**

**Figure S1. Pairwise distance matrix of ITS sequences, based on Jukes-Cantor, of *Croton* *buiquensis* and species of section *Pedicellati*.**
